# Psychopathology Changes in Alzheimer’s Disease

**DOI:** 10.3390/bs12110418

**Published:** 2022-10-28

**Authors:** Joana Henriques-Calado

**Affiliations:** 1 Faculdade de Psicologia, Universidade de Lisboa, Alameda da Universidade, 1649-013 Lisboa, Portugal; jhcalado@psicologia.ulisboa.pt; 2 CICPSI, Faculdade de Psicologia, Universidade de Lisboa, Alameda da Universidade, 1649-013 Lisboa, Portugal

**Keywords:** Alzheimer’s disease, psychopathology, personality disorders, personality, aging

## Abstract

The advances in knowledge about the association between personality and neuropathology in Alzheimer’s disease have been highlighted. This research is oriented to the evaluation of personality changes in the screening of axis II personality disorders in Alzheimer’s disease. The investigation was managed with four groups to whom were applied the PDQ-4+ in individual interviews. Some results are in line with the state-of-the-art review and we also provide new research data. A higher global personality disorder index and greater incidence of clusters C (anxious) and A (odd/eccentric) are confirmed as personality changes. Interpretive possibilities of the data and their implications for the study of psychopathology changes in Alzheimer’s disease are discussed.

## 1. Introduction

The advances in knowledge about the association between personality and neuropathology in Alzheimer’s disease (AD) is highlighted in the literature [1,2,3,4]. In turn, personality changes in AD have been documented in the literature and may be a useful early clinical marker [5,6,7,8,9,10,11,12,13,14,15,16,17,18].

It has been suggested that the pre-morbid characteristics of personality may represent a risk factor for AD and should differ between patients and controls [17,19]. Prospectively, personality assessment is suggested to be included in the diagnosis, having important implications for the prevention and treatment of symptoms and for the etiological knowledge of dementia [7,8,15,16,19]. Vast research data have shown that some pre-morbid personality characteristics even play a role in modifying the disease process or its phenotypic expression, as an example in the manifestation of the behavioral and psychological symptoms of dementia [17,20]. Nevertheless, the link between premorbid personality/personality disorders as potential risk factors for AD has been much less well-informed [20,21,22,23,24,25,26,27,28,29,30].

It should be noted that there are references to AD in the literature that point to the evidence of associations between psychopathological signs and symptoms and personality disorders. However, few studies have been designed to specifically address this issue through research into personality disorders (DSM-Axis II) from the perspective of premorbidity and the clinical state of AD and also personality psychopathology changes. This paper proposes to reflect upon these hypotheses.

### Aim of the Study

As a proposal, it is intended to clarify the impact of personality disorders on AD, through the PDQ-4+, by investigating whether personality psychopathology remain stable or undergo changes. The personality disorders changes will be analyzed as a product of the difference between current and premorbid personality traits. The following hypothesis is studied: regarding personality psychopathology changes, the Alzheimer´s Disease group is expected to show a significant increase in the mean result in the global personality disorder index (PDQ-4+ Total) and a significant increase in clusters B/C and A [31,32,33,34,35,36], in comparison with the data gathered from the Alzheimer´s Disease group informants and the control groups.

## 2. Materials and Methods

### 2.1. Participants

Regarding the clinical group, the Alzheimer´s disease (AD) group is organized by 44 females, aged 65 years or above, Caucasian participants of Portuguese nationality, living in an urban environment, with a clinical diagnosis of AD (onset) (*M*_Age_ = 81.36 years, *SD* = 6.47 years), with an average of 7.61 years of schooling (*SD* = 4.00 years), and an average of 17.59 points (*SD* = 4.44) in the MMSE.

Regarding the control group, it comprises 80 females, aged 65 years or above, Caucasian participants of Portuguese nationality, living in an urban environment (*M*_Age_ = 75.84 years, *SD* = 6.12 years), with an average of 8.94 years of schooling (*SD* = 2.75 years), and an average of 27.81 points (*SD* = 2.08) in the MMSE.

The Alzheimer´s disease (AD) Group informants and the control group informants are organized by 40 and 42 relatives, respectively, who provide assessments of the pre-morbid personality characteristics.

For the AD Group and the control group, possible correlations between the clinical variable of the MMSE Total and the Age and Schooling variables were evaluated, verifying that, was neither observed to be related to Age (*r* = −0.07, *p* = 0.67) nor Schooling (*r_s_* = 0.16, *p* = 0.29) in the AD group, and also, in the control group was neither found to be related to Age (*r* = −0.32, *p* = 0.11) nor Schooling (*r_s_* = 0.35, *p* = 0.08).

### 2.2. Measures

#### 2.2.1. Socio-Demographic Questionnaire (e.g., Age, Schooling)

#### 2.2.2. Mini Mental State Examination (MMSE)

The MMSE is an instrument consisting of 30 items that allows access to a total score, being widely used in clinical and research contexts to measure cognitive impairment.

#### 2.2.3. The Personality Diagnostic Questionnaire (PDQ–4+)

The Personality Diagnostic Questionnaire 4+ [37] is a self-report questionnaire with 99 items based on true/false answers, designed to generate diagnoses that are compatible with the diagnostic criteria of the DSM-IV Axis II for personality disorders. The PDQ-4+ assesses the ten personality disorders (scales) and respective clusters included in the DSM-IV [38] (Cluster A—Paranoid, Schizoid, Schizotypal; Cluster B—Histrionic, Narcissistic, Borderline, Antisocial; Cluster C—Avoidant, Dependent, Obsessive-compulsive) and a further two personality disorders which appear in the DSM-IV in Appendix B (Negativistic and Depressive). Additionally, the questionnaire introduces the analysis for a global personality disorder index (PDQ-4+ Total), which is determined on the basis of the score of all the positive results. Studies show that PDQ-4+ Total ≤ 20 points: without evidence of personality disorder/“normal” control groups; PDQ-4+ Total ≥ 30 points: high likelihood of evidence of significant personality disorder [37].

Likewise, a version of the PDQ-4+ for informants was introduced, along with the same method used by other investigations in this context [12,13,17,18,31,37,39,40]. With the specific aim of retrospectively questioning the informant’s relative, the instruction is as follows: “Think of your relative before the age of 60 years. Remember what she was like in the past, throughout her whole life, and answer the following questions”—this methodology is based on other works [12,13,17,18]. 

The PDQ-4+ has proven to serve reasonably well as a screening instrument since it is able to adequately indicate the absence of a disorder, while its use is strongly recommended for triage/screening and serves as a provisional personality disorder diagnosis in clinical and non-clinical samples [37,41].

The analysis of the PDQ-4+ reliability by calculating the *Kuder-Richardson Formula 20* (α) [37] show: AD group—PDQ-4+ Total (α 0.94), Cluster A (α 0.82), Cluster B (α 0.88), Cluster C (α 0.82), (Appendix B α 0.77); Control Group—PDQ-4+ Total (α 0.90), Cluster A (α 0.77), Cluster B (α 0.78), Cluster C (α 0.77), (Appendix B α 0.61); AD Group Informants—PDQ-4+ Total (α 0.92), Cluster A (α 0.69), Cluster B (α 0.88), Cluster C (α 0.79), (Appendix B α 0.69); Control Group Informants—PDQ-4+ Total (α 0.88), Cluster A (α 0.62), Cluster B (α 0.77), Cluster C (α 0.74), (Appendix B α 0.70).

Direct and high correlations are observed in the analysis performed on all the samples of the pattern of correlations between the scales/clusters and the PDQ-4+ Total, which reveal and confirm the reliability and validity of the instrument. 

### 2.3. Procedure

This research received approval and authorization from the Ethics Committee of the affiliated institution and by the host institutions. The study was conducted in accordance with the Declaration of Helsinki and in compliance with the recommendations stipulated by Alzheimer Europe [42].

#### 2.3.1. AD Group and AD Group Informants

The collection of the sample of AD group took place at a Psychiatric Hospital Center (±69%) and at Geriatric Centers (±31%). 

The following inclusion criteria were taken into account: female; 65 years or older; clinical diagnosis of AD (onset); absence of psychiatric or neurological comorbidity; with abilities of intelligibility and interpersonal relationships. It is noteworthy that the AD clinical diagnosis considered the medical evaluation as a criterion [37,41,43,44].

Towards the AD group informants, each member was a relative in a close relationship with the participant. 

As regards the groups, the protocol was conducted in a face-to-face individual session by a psychologist.

#### 2.3.2. Control Group and Control Group Informants

The collection of the sample of the control group sample took place at a day center (19 participants) and by means of a “snowball” collaboration (61 participants).

The following inclusion criteria were taken into account: female; 65 years or above; from the general population; absence of diagnosed or evident psychiatric or neurological disorder; with abilities of intelligibility and interpersonal relationships.

As regards collection from the control group and the application protocol were conducted as the previously described situation.

### 2.4. Data Analysis

Data relative to the PDQ-4+ Total and data relative to the three clusters of the PDQ-4+ (Cluster A, Cluster B, Cluster C) will be analyzed. The ten personality disorder scales of the PDQ-4+ will be considered for a secondary analysis. Globally, these options stem from the empirical research of the instrument, the literature review, and the study of the adapted version of the instrument to this study. It is a recommended evaluation instrument and used in the form of triage/screening for personality disorders, as a provisional diagnosis or indicator of a personality disorder [37,41].

The PDQ-4+ Total is a quantitative variable. The clusters will be evaluated from a categorical or metric perspective, according to the aim, whereas the scales will be analyzed as the absence or presence of a personality disorder diagnosis (0 or 1). For each example, for true response (1) the scorer should find the diagnostic criteria for the specific diagnosis that the item assesses on the scoring key, then they should check if off on the score sheet. If the threshold is reached or exceeded (as a score of four or more paranoid items would indicate), the diagnosis is recorded [37].

## 3. Results

### 3.1. Current and Premorbid Psychopathology Studies

In an initial analysis to assess whether there is a significant influence of the groups (AD group informants and the AD group) on the global personality disorder index (PDQ-4+ Total), a one factor analysis of variance was carried out—ANOVA. The assumptions of this statistical method were validated—normality and homogeneity of variances: *Levene* (*p* = 0.45). The results are presented in Table 1.

In accordance with the test, the difference observed is not statistically significant for the PDQ-4+ Total. In comparison with the AD group, the AD group informants do not present a significantly higher mean result of the PDQ-4+ Total.

When the cut-off score of the PDQ-4+ Total is applied, through the equality of means test (Student’s t-test) for independent samples with validation of the respective assumptions, the AD group is found to have significantly higher mean scores than the AD group informants when PDQ-4+ Total ≥ 30 points, thus differentiating the groups from each other when PDQ-4+ Total < 20 points (*t*(4) = 0.26, *p* = 0.81, *η*^2^*_p_*= 0.02).

**Figure 1:**
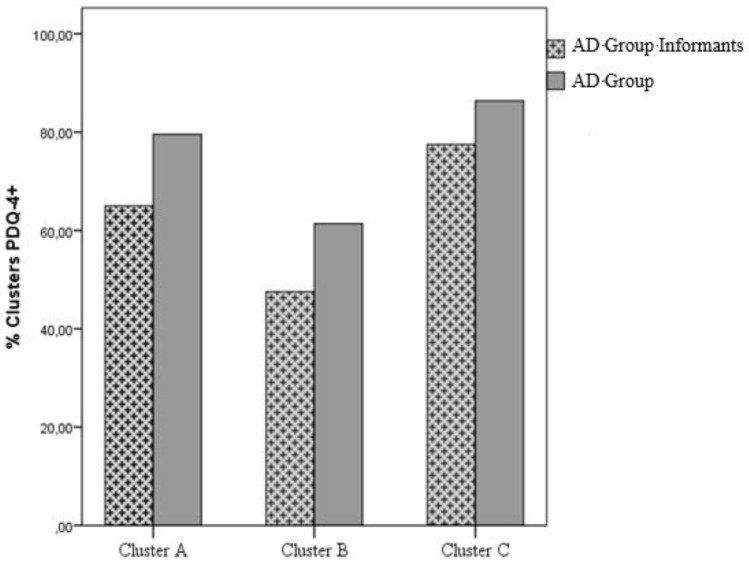
Bar graph of the percentage distribution of the three PDQ-4+ clusters in AD Group Informants and AD Group.

In an initial analysis to assess the statistical significance of the different incidence of diagnoses in each cluster of the PDQ-4+ in the groups (AD group informants and AD group), the Wilcoxon-Mann–Whitney test was performed (Table 2; Figure 1).

**Figure 2:**
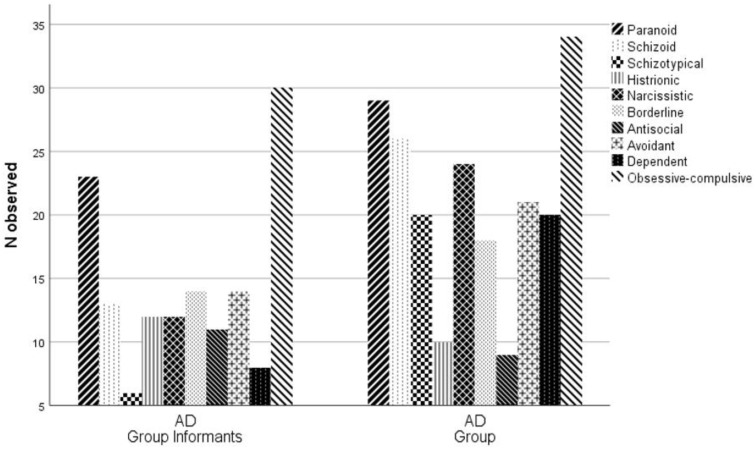
Bar graph of the observed distribution of the ten scales of the PDQ-4+ in AD Group Informants and AD Group.

In accordance with the tests, the differences observed in the incidence of the clusters of the PDQ-4+ are statistically significant for one of the clusters. In comparison with the AD group informants, the AD group presents a significantly higher incidence of diagnoses relative to cluster A. Secondarily, in order to assess the statistical significance of the different incidence of a personality disorder diagnosis for each scale of the PDQ-4+ in the groups (AD group informants and AD group), a Chi-Square Test of Homogeneity was performed (Table 3; Figure 2).

In accordance with the tests, the differences observed in the incidence of the personality disorder scales of the PDQ-4+ are statistically significant for the Schizoid, Schizotypal, Narcissistic and Dependent scales. In comparison with the AD group informants, the AD group is observed to have a significantly higher incidence of these diagnoses.

### 3.2. Psychopathology Changes Study

The next main objective was to assess whether the results observed in the mean results of PDQ-4+ Total and in clusters A, B and C of the PDQ-4+, were of statistical significance for psychopathology changes. A new variable related to the difference between the current and pre-morbid personality was created by the PDQ-4+ mean data: Personality Changes AD Group (PCADGroup) and Personality Changes Control Group (PCCGroup).

With the objective of analyzing the influence of the PCADGroup and PCCGroup on the PDQ-4+ changes, stemming from the difference between the current and pre-morbid personality, an ANOVA was carried out. The normality and homogeneity of variances were validated: *Levene* (*p* ≥ 0.11 for all variables; except for Cluster B which presents heterogeneous variance, *p* ≤ 0.001). The Welch’s F test, the alternative statistical approach to ANOVA, was used for this dimension with heterogeneous variance. The results are presented in Table 4.

In accordance with the tests, the differences observed are statistically significant for three variables of the PDQ-4+ and the effect size varies between small and average. In comparison with the PCCGroup, the PCADGroup presents significant differences, and one highly significant difference, in the variables of PDQ-4+. The PCADGroup reveals significant personality changes in comparison with the PCCGroup, stemming from the difference in the mean results of the variables of the PDQ-4+ between current and pre-morbid personality, in particular a rise in the PDQ-4+ Total and in clusters A and C.

## 4. Discussion

In terms of changes in personality disorders screening (DSM-IV-Axis II), a significantly higher mean result in the global personality disorder index (PDQ-4+ Total) and rise incidence of clusters C and A in the AD group in comparison with the control group are confirmed. It is a partial confirmation of hypothesis since no significant increase in the incidence of cluster B is observed in the AD group. An analysis and suggestions for understanding the data will now be presented.

Let us begin with an initial comparative analysis. The AD group (current personality measurement) does not demonstrate a higher mean result in the global personality disorder index (PDQ-4+ Total) in comparison with the information collected from the AD group informants (pre-morbid personality measurement). When the cut-off score of the PDQ-4+ Total ≥ 30 points [37] is applied to both groups, a strong likelihood of the evidence of significant personality disorder is identified. Taking this into consideration, and focusing on this comparative analysis, one may perhaps conclude that despite an increase in the psychopathological symptomatology of dementia, the latter is not significant and no differences in the global personality disorder index are observed. However, there does seem to be evidence of continuity between possible pre-morbid psychopathology and the present moment in AD. In this initial comparative analysis, the AD group presents a higher incidence of cluster A (odd/eccentric) in comparison with the AD group informants. Hence, a higher incidence of cluster A at the present moment of Dementia is observed when compared with its pre-morbidity. Furthermore, in both pre-morbidity and at the present moment, individuals with AD reveal a pattern of possible personality disorder in the following order of magnitude: Cluster C, Cluster A, and Cluster B. Consequently, the individuals in this study with AD were found to have maintained a particular pattern (regardless of the variations), particularly the tendency to frequently appear anxious, fearful, and dependent (Cluster C) and, finally, to frequently appear dramatic, emotional, or inconstant (Cluster B) [38]. Some results are in line with the state-of-the-art review [31,32,33,34,35,36].

However, when the differences observed in the results of the PDQ-4+ of the main analysis are evaluated, namely those which stem from the difference between the current and pre-morbid personality, referred to as the Personality Changes AD Group and the Personality Changes Control Group, personality changes are observed. These changes are reflected in an increase in the global personality disorder index (PDQ-4+ Total) and in clusters A and C, with an effect size that varies between small and average. No change is observed for cluster B. These data are in line with the discussion of the previously analyzed points of this section. Nevertheless, the possibility of cluster B being characteristic of behavioural functioning across the life course of individuals with AD should also be noted. As far as the magnitude of the personality disorders is concerned, they were observed in the following decreasing order: increase in cluster A incidence followed much later by cluster C and an increase in the global personality disorder index (PDQ-4+ Total).

According to the analysis presented in this study and comparisons between self-reports and informants, there appears to be evidence of a continuity in the incidence of cluster B personality disorders and personality changes, reflected in an increase in the global personality disorder index (PDQ-4+ Total) and the incidence of clusters A (odd/eccentric) and C (anxious).

In an exploratory analysis of the personality disorder scales, an increase in some disorders may be observed between a pre-morbidity state and a state of dementia, namely, by order of incidence: schizoid, narcissistic, dependent, and schizotypal. This observation is not meant to be taken as a diagnosis, but rather as an indicator or tendency. So, individuals with AD currently present with an increased display of these personality deviations in comparison with their pre-morbidity state.

Limitations: the small size of the samples, although this reflects the difficult access to participants diagnosed with AD in its early stages and also informants available to participate. It should also be noted that the retrospective evaluation of proxies can introduce fallacies.

Finally, developing new studies which take these pathological personality variables and relations into consideration appears to be important for the possible introduction of personality evaluation (abnormal personality dimensions) in the diagnosis of AD and follow-up assessments, and perhaps even as a tool to control the efficacy of therapeutic treatment.

## Figures and Tables

**Table 1:** Result of the Variance Analysis (ANOVA) of the AD Group Informants and the AD Group on the Global Personality Disorder Index (PDQ-4+ Total).

	AD Group Informants (*n* = 40)	AD Group (*n* = 44)				
Variable	*M (SD)*	*M (SD)*	*F*	*p*	*η* ^2^ *_p_*	*π*
PDQ-4+ Total	38.50 (15.16)	44.57 (17.41)	2.87	0.09	0.03	0.39

*Note*. *η*^2^*_p_* (effect size): ≤ 0.05 (Small); ] 0.05; 0.25] (Medium); ] 0.25; 0.50] (High); > 0.50 (Very high); π (test power): ≥ 0.80; 1:00] [45].

**Table 2:** Results of the Wilcoxon-Mann–Whitney Test between the AD Group Informants and the AD Group on the Incidence of Clusters of the PDQ-4+.

	AD Group Informants (*n* = 40)	AD Group (*n* = 44)			
Clusters	% (*n* Observed)	% (*n* Observed)	*U*	*Z*	*p*
Cluster A	65.00 (26)	79.55 (35)	594.50	−2.64	**0.008**
Cluster B	47.50 (19)	61.36 (27)	802.00	−0.74	0.46
Cluster C	77.50 (31)	88.06 (39)	700.00	−1.68	0.09

*Note*. In bold are identified cases in which *p* < 0.05.

**Table 3:** Chi-square Test of Homogeneity between the AD Group Informants and the AD Group for Diagnosis of the Ten Scales of the PDQ-4+.

	AD Group Informants (*n* = 40)	AD Group (*n* = 44)			
PDQ-4+ Scales	% (*n* Observed)	% (*n* Observed)	χ^2^	df	*p*
Paranoid	57.50 (23)	65.90 (29)	0.32	1	0.57
Schizoid	32.50 (13)	59.10 (26)	4.94	1	**0.03**
Schizotypical	15.00 (6)	45.50 (20)	7.72	1	**0.005**
Histrionic	30.00 (12)	22.70 (10)	0.26	1	0.61
Narcissistic	30.00 (12)	54.50 (24)	4.20	1	**0.04**
Borderline	35.00 (14)	40.90 (18)	0.11	1	0.74
Antisocial	27.50 (11)	20.50 (9)	0.25	1	0.62
Avoidant	35.00 (14)	47.70 (21)	2.88	1	0.24
Dependent	20.00 (8)	45.50 (20)	5.02	1	**0.03**
Obsessive-Compulsive	75.00 (30)	77.30 (34)	0.001	1	1.00

*Note*. In bold are identified cases in which *p* < 0.05.

**Table 4:** Analysis of Variance (ANOVA) of the Personality Changes AD Group (PCADGroup) and the Personality Changes Control Group (PCCGroup) on the PDQ-4+ between Current and Pre-morbidity.

	PCADGroup (*n* = 40)	PCCGroup (*n* = 42)				
≠ PDQ-4+ Dimensions Current–Pre-Morbid	*M (SD)*	*M (SD)*	*F*	*p*	*η* ^2^ *_p_*	*π*
PDQ-4+ Total	6.52 (21.68)	−2.90 (18.31)	4.54	**0.04**	0.05	0.56
Cluster A	0.78 (1.35)	−0.43 (1.47)	14.91	**0.001**	0.16	0.97
Cluster B ^a^	0.28 (1.78)	−0.19 (0.99)	2.13	0.15	0.03	0.31
Cluster C	0.38 (1.41)	−0.26 (1.11)	5.22	**0.03**	0.06	0.62

*Note*. In bold are identified cases in which *p* < 0.05; ^a^ Welch’s F statistic.

## Data Availability

The datasets used and/or analysed during the current study are available from the corresponding author on reasonable request.
